# Carbon-11 Production: Communication, Operations, Maintenance, Troubleshooting, and Analysis for Maintaining High-Grade Bombardment and Provisions of [^11^C]Carbon Dioxide and Its Conversion to [^11^C]Methyl Iodide

**DOI:** 10.3390/molecules31122095

**Published:** 2026-06-15

**Authors:** Simon K. Joseph, Andrew Tavare, Kiara Thomas, Dae-In Kim, Kaleigh Timmins, Melchor V. Cantorias, Briana Roman, Jakub Mroz, Jairo Baquero, Julian Calderin, Lucas Fernandez, Sandy Phung, Andrew Chung, Patrick Carberry

**Affiliations:** 1 Department of Radiology, New York University Grossman School of Medicine, 660 First Avenue, Room 240, New York, NY 10016, USA; simon.joseph@nyulangone.org (S.K.J.); kiara.thomas@nyulangone.org (K.T.); kaleigh.timmins@nyulangone.org (K.T.); melchor.cantorias@nyulangone.org (M.V.C.); briana.roman@nyulangone.org (B.R.); jakub.mroz@nyulangone.org (J.M.); jairo.baquerobuitrago@nyulangone.org (J.B.); 2 Siemens Healthineers (PETNET SOLUTIONS NYU), 660 First Avenue, Room 140, New York, NY 10016, USA; andrew.tavare@petnetsolutions.com (A.T.); julian.calderin@petnetsolutions.com (J.C.); lucas.fernandez@siemens-healthineers.com (L.F.); sandy.phung@petnetsolutions.com (S.P.); andrew.chung@petnetsolutions.com (A.C.); 3 Radiation Safety, Real Estate Development and Facilities, NYU Langone Health, Berkley Park, 339 E. 28th St, New York, NY 10016, USA; dae.inkim@nyulangone.org

**Keywords:** carbon-11, cyclotron, [^11^C]carbon dioxide, [^11^C]methyl iodide, target, radiochemistry, positron emission tomography, molar activity, isotopologue mass

## Abstract

Incorporation of carbon-11 radiotracers for positron emission tomography (PET) imaging requires close coordination between cyclotron operation, radiochemistry production, quality control, and clinical administration. A persistent challenge exists is the minimization of the carbon-12 isotopologue mass of the radiotracer, which reduces molar activity and can compromise PET image quality. This challenge can be particularly acute at facilities where cyclotron operation and carbon-11 radiochemistry are realized by separate organizations with distinct operational priorities. Here, we describe how the Radiochemistry Group at New York University Grossman School of Medicine and Siemens Healthineers have developed an integrated operational framework for consistent, high-quality carbon-11 production within an academic–industry partnership. Cyclotron target maintenance and conditioning protocols, remote chemistry module maintenance schedules, a validated radio-HPLC method (UV LOD = 0.9 µg/mL, UV LOQ = 3.0 µg/mL) for trending methyl iodide isotopologue mass, and structured inter-team communication protocols are presented in this manuscript. Quality analysis demonstrates molar activities consistently exceeding the recommended minimum of 40 GBq/µmol for reversibly binding radiotracers used in human PET studies. This work is intended as a practical resource for radiochemists, cyclotron engineers, and facility managers working to establish or improve institutional carbon-11 programs.

## 1. Introduction

The cyclotron, a circular particle accelerator invented by Ernest Lawrence at the University of California, Berkeley in the 1930s [1,2,3], remains a primary tool for producing radioisotopes used in positron emission tomography (PET) imaging [4,5]. When paired with computed tomography (CT) [6] or magnetic resonance imaging (MRI) [7], PET is a dual-modality imaging technique that can track a wide range of physiological and biological processes on the molecular scale. Prominent PET isotopes generated via cyclotron include carbon-11 [8,9], fluorine-18 [10], nitrogen-13 [11], and oxygen-15 [12]. These radionuclides serve as the radiolabel in a particular radiochemical synthesis, which yields a radiopharmaceutical or radiotracer for use in clinical diagnostics or research [13,14].

**Figure 1:**
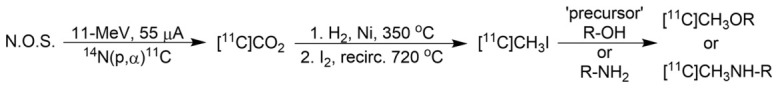
Cyclotron bombardment and formation of [^11^C]CH_3_I used in carbon-11 production.

Carbon-11 is a short-lived positron-emitting radionuclide with a half-life of 20.4 min. Exchanging a carbon-12 atom for a carbon-11 radionuclide affords a PET probe that maintains the chemical characteristics of the reference molecular entity, allowing for direct in-vivo or ex-vivo examination of its equivalent physiological relationship with a biological target of interest. Proton bombardment of a cyclotron with hydrogen (5–10%) as the target gas provides [^11^C]methane ([^11^C]CH_4_) as the starting material for radiolabeling [8,15]. The primary method of carbon-11 production is by the bombardment of high-purity nitrogen gas (0.5–5% oxygen in nitrogen) with the use of a cyclotron via a nuclear reaction to provide [^11^C]carbon dioxide ([^11^C]CO_2_) gas [16]. Currently, most routinely produced carbon-11 radiotracers employed in research studies involve radiosyntheses that utilize the transformation of this [^11^C]CO_2_ into the more reactive electrophilic precursor, carbon-11 methyl iodide ([^11^C]CH_3_I) [17,18], as depicted in Figure 1. In a demonstration of control, consistency, reproducibility, and safety, which are the inherent requirements for a study application to the Food and Drug Administration (FDA) under the Investigative New Drug (IND) pathways or to internal Radioactive Drug Research Committees (RDRCs), most developed synthesis protocols for routine radiotracer provisions are subsequently validated on remote chemistry platforms [19] for ultimate dispensing to human research PET studies.

There are several challenges in successfully providing carbon-11 radiotracers for administration in research studies. Due to its short half-life, expiry times are limited to 1–2 h post-end-of-synthesis (EOS), with up to 30 min of time devoted to quality control testing and release of the radiotracer. Transportation of the radiopharmaceutical is extremely limited, requiring that the PET clinic, cyclotron, and radiochemistry facility are all under the same roof or, at minimum, in proximity. 

Another challenge is the lower limit of activity necessary for a useful PET scan. The typical range of activity administered to an average-sized adult [20] is about 185–740 MBq (5–20 mCi). This poses a major hurdle for most sites due to the conversion of [^11^C]CO_2_ to [^11^C]CH_3_I, which has an acceptable conversion efficiency of 37 ± 7% from a major manufacturer of automated modules platforms [21] or with the use of the “wet” method to convert [^11^C]CO_2_ into [^11^C]CH_3_I by way of lithium aluminum hydride and hydroiodic acid [22,23] with typical synthesis times required for radiolabeling and purification/formulation on the order of 30–50 min (e.g., 1–2 half-lives). A further constraint is the tolerable isotopologue mass of the carbon-12 form of the radiotracer, which is typically ten-to-three hundred-fold below the no observed adverse effect level (NOAEL) [24,25,26]. The allowable limit for carbon-12 impurities may be of the order of 10-fold less per dose. This, in turn, may lead to volume restrictions of injectable radiotracers and can have a negative effect on the quality of the reconstructed PET images.

The carbon-12 counterpart of the final radiotracer becomes present in a manufacturing process related to the quantity and purity of formed [^11^C]CH_3_I from its conversion on a synthesis module and from the quality of generated [^11^C]CO_2_ produced and delivered by the cyclotron. Molar activity (A_m_) is defined as the amount of radioactivity over the molar sum of all radioisotopes from the desired compound using Equation (1):

(1) $$A_{m}\;=\;\frac{A_{i}}{n_{i}}$$

where A*_i_* is the activity of the radiopharmaceutical, expressed in GBq, and n*_i_* is the molar amount (µmol) of the sum of all forms, radioactive and stable nuclides, of the pharmaceutical; for carbon-11 radiotracers, this amount is predominantly derived from the isotopologue carbon-12 form of the radiopharmaceutical, although the carbon-13 and carbon-14 isomers cannot be ignored [27]. In this manuscript, only the carbon-12 isotopologue form is used in the calculations. The SI unit for expressing A_m_ of a carbon-11 radiopharmaceutical is GBq/µmol [28].

Binding potential (*BP*), a key outcome measurement in reversible-binding PET, is directly affected by molar activity. The relationship described by *BP* and target volume of distribution (V_T_) requires that the free radioligand concentration be significantly lower than the equilibrium constant (K*_D_*), and that the concentration of bound radioligand (B) be significantly lower than total receptor density (B*_max_*), conditions which are satisfied by the utilization of radiotracers of high molar activity [29,30]. A study considering the implications of A_m_ on the binding potentials of [^11^C]PiB scans in amyloid precursor protein transgenic (Tg) mice found there to be a significant association between attenuation of binding potential and decreasing A_m_ values from 200 GBq/µmol to 20 GBq/µmol despite administering the same radioactive dose strength [31]. As such, the capability of PET to provide valid quantitative measures of the nanomolar concentrations of low-density protein distribution is predicated on the high molar radioactivity of the radioligand probe. It is recommended that reversibly binding radiotracers used in humans carry a molar activity of at least 40 GBq/µmol at the time of administration, with higher values desirable [32].

In PET imaging, minimizing the amount of carbon-12 isotopologues of the radiotracer is essential to avoid interference with the body’s natural physiological functions. A larger quantity of carbon-12 compound indicates a greater injection of the non-radioactive chemical, which may cause toxicity or receptor blockage (saturation). The objective is to maximize the proportion of carbon-11 radiotracers relative to the carbon-12 isotopes to achieve a high molar activity. Excessive carbon-12 isotope reduces molar activity, potentially resulting in low-quality medical images. These factors significantly impact both research efforts and operational goals in PET imaging.

The importance of high molar activity extends beyond clinical imaging to radiotracer development itself. The assessment of candidate PET probes requires accurate measurement of parameters such as affinity (1/K_D_, 1/K*_i_*), lipophilicity (LogD_7.4_), binding potential (B*_max_*/K_D_ (or K*_i_*)), and metabolic fate [33,34,35,36]. Measurements of pharmacokinetic characteristics can be confounded or invalidated under the condition of low radiotracer A_m_ [37]. Unreliable molar activity therefore compromises not only the quality of clinical PET images but also the upstream science of radiotracer selection [32]. The swath of eligible molecules for carbon-11 radiolabeling is profoundly vast, and the approaches for introduction of the radiolabel, whether by heteroatom alkylation, carbon–carbon methylation, [^11^C]CN cyanation, [^11^C]CO_2_ fixation, or [^11^C]CO carbonylation, are contingent on the effective and reliable provisions of high-quality [^11^C]CO_2_, from which they ultimately derive [8,38,39,40,41].

Yet while individual aspects of carbon-11 production—including the cyclotron, cyclotron targets, automated synthesis, and quality control—have been described in the literature, comprehensive operational guides that integrate these elements with radiation safety compliance and inter-team communication protocols remain scarce. This manuscript aims to address that gap.

The facility at New York University Grossman School of Medicine (NYUGSoM) houses a Siemens Eclipse HP (11 MeV) cyclotron with two dedicated carbon-11 targets for generating [^11^C]CO_2_ via proton bombardment of 1% oxygen/99% nitrogen (N.O.S.) gas [42]. The cyclotron is operated and maintained by Siemens Healthineers; production of carbon-11 research doses for human injections are performed by the NYUGSoM Radiochemistry Group. In this manuscript, we describe how this academic–industry partnership maintains consistent, high-quality [^11^C]CO_2_ and [^11^C]CH_3_I production through coordinated cyclotron target maintenance, automated module upkeep, analytical monitoring of isotopologue mass and molar activity, radiation safety compliance, and structured inter-team communication protocols.

## 2. Results and Discussions

### 2.1. Cyclotron and Operations

**Figure 2:**
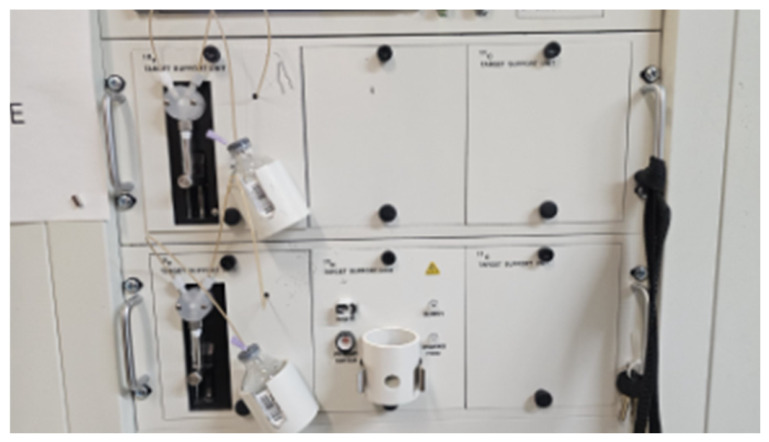
Target support unit (TSU).

At NYUGSoM, the cyclotron employed for human and animal radioisotope research production is an 11-MeV Siemens Eclipse HP for producing [^11^C]CO_2_ via ^14^N[p,α]^11^C. This system is a self-shielded negative-ion particle accelerator with dual beam extraction, consisting of a vacuum system, an ion source with radio frequency (RF) acceleration, a magnet, and a target system. The Eclipse HP also includes a water-cooling cabinet for the cooling system, an electrical cabinet, and a target support unit (TSU) for reagent or gas distribution to designated target (see Figure 2).

A target support unit (TSU) is used for all cyclotron targets, mechanically and electrically separating the target system from the cyclotron (see Figure 2). TSU supplies replenishable materials to the target for irradiation and recovers the resulting radionuclide at the end of irradiation. TSU controls and delivers the inert gas used in delivering raw radionuclides from the bombarded target to chemical synthesis units. Target loading and unloading are done automatically under computer control. The procedure differs according to the type of target and particular mode of delivery required.

**Figure 3:**
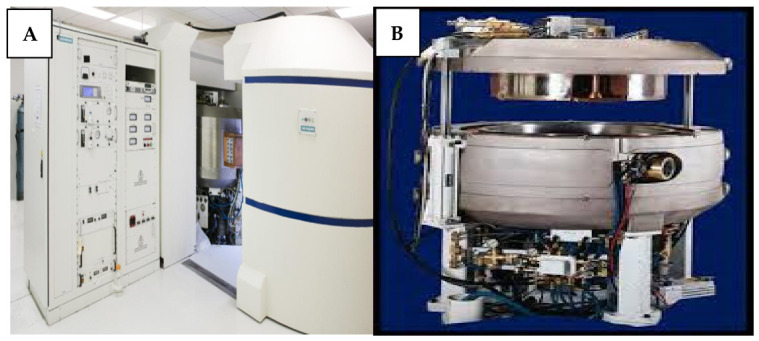
(**A**) Siemens Eclipse HP cyclotron (**B**) and cyclotron target area and vacuum tank.

The cyclotron shielding door is cast in interlocking blocks of polyethylene, boron carbide cement, and lead pellet beads (see Figure 3A). These shields can be opened to provide access to the target area and cyclotron vacuum tank (see Figure 3B). The cyclotron has two main shielding doors, one adjacent to each side of beamlines 1 and 2. The radiation dose outside the shield is less than 2.5 mRem per hour, averaged over the perimeter while bombarding any target at the maximum target current of 60 µA. Safety and survey area checks are conducted on a maintenance schedule by Siemens to ensure permission for the beam to be on while the shielded doors are open.

**Figure 4:**
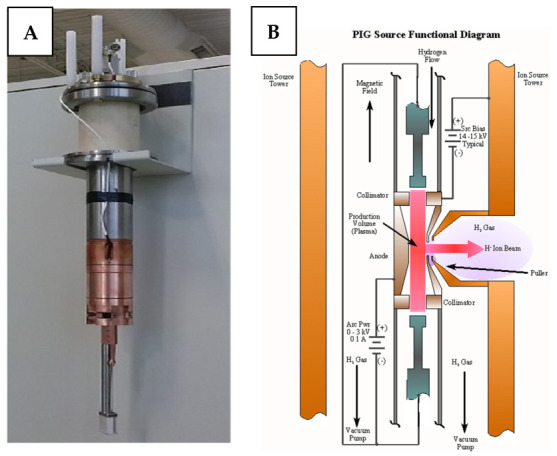
(**A**) Ion source and tower and (**B**) ion source schematics.

**Figure 5:**
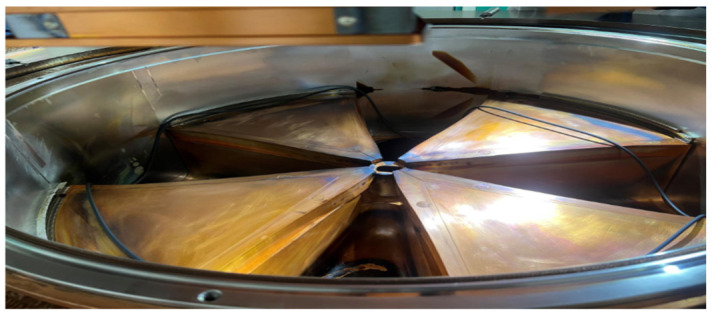
Dees, valley, central region, and magnetic field.

The Siemens Eclipse HP uses a Penning ion gauge (PIG) ion source for creating the beam (see Figure 4A). The ion source operates by ionizing hydrogen to produce negative ions (H^−^) and is sourced from 99.9999% purity hydrogen gas. The ion source power supply is a 1 Amp supplier, designed for running cyclotron target operation currents up to 120 µA for dual bombardment. The PIG source generates negative hydrogen ions (H^−^) in a central region, which are accelerated through four dee electrodes under radio-frequency oscillation and a confining magnetic field. At the extraction radius, carbon stripping foils convert the H^−^ ions to protons (H^+^), which are directed through the beam tube to the target for bombardment (see Figure 5). The ion source schematic is such that the ions are extracted by a set of cathodes and collimators being pulled through an anode slit by an electric field causing a beam to be pulled by the tower puller creating a beam (see Figure 4B).

Common issues that can occur with this type of ion source include cathode sputtering, causing blocking of the beam; no beam on post; no beam for acceleration; and high ion source output power during dual-beam operation. If any of these issues occur, a rebuild of the ion source or tower is needed (see Supplementary Data, Section S2). Both the NYUGSoM Radiochemistry Group and Siemens Healthineers monitor the ion source usage and current parameters to avoid stressing the cyclotron. Reducing the beam current is a common operational strategy used to extend the lifespan of critical internal components, particularly the ion source and the extraction of foils and targets.

**Figure 6:**
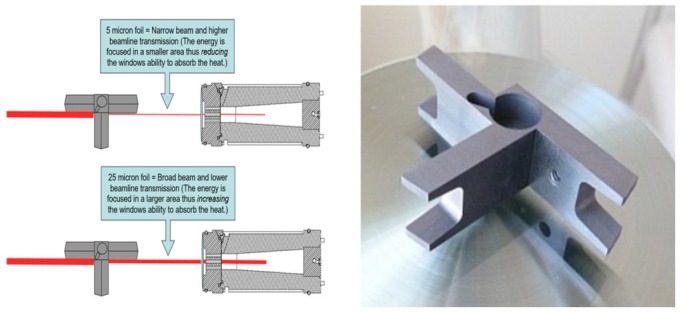
Extractor holds the 3-foil used to bombard the target, 5– and 25–micron foil size.

Carbon stripping foils held by the extractors are used to convert H^−^ to protons (H^+^) for target bombardment. The extractor foils have three positions with different ranges, predetermined by computer settings. Carbon-11 targets use a 25-micron-thick foil to disperse the beam enabling a larger surface area to bombard the mixed N.O.S. gas (see Figure 6). When the carbon-11 target is placed in position, the computer program automatically switches the extractor foil to position 3 for the correct 25-micron thickness. Use of the wrong size foil may cause a pinhole leak in the target Havar window, creating potential vacuum leaks, loss of foils, and ion gauge filament failure. Ensuring the correct setup before bombardment is vital to prevent accidental exposure or target rupture due to improper loading. The teams repeat and confirm that the correct target and foil are positioned before proceeding. The operation transmission percentage range for extraction on the foils is 60–75%. Transmission above 75% may cause higher yield but the beam size is thinner and will cause a leak to the target’s Havar window causing a leak (see Figure 2, Figure 3, Figure 4, Figure 5 and Figure 6).

**Figure 7:**
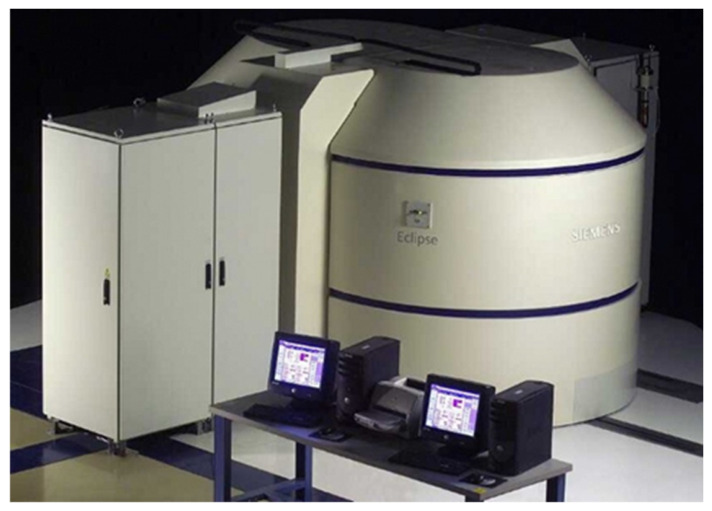
Primary computer and secondary computer.

The cyclotron is controlled by a dedicated computer station using programming logic and control electronics. The user interface and graphical user interface (GUI) operate on two terminals: the primary computer (main computer) and the secondary computer (satellite terminal) (see Figure 7). The cyclotron suite has full control of the cyclotron, and the radiochemistry group has view-only access. Siemens Healthineers maintain strict oversight of the cyclotron to ensure safety and regulatory compliance of their protocols. Both groups monitor the parameters of the cyclotron groups during bombarding.

### 2.2. Carbon-11 Target Removal and Cleaning

At NYUGSoM, the cyclotron follows a procedure for cleaning and servicing the carbon-11 aluminum HP target assembly for the Siemens Eclipse HP. This procedure includes purging, removal, disassembly, cleaning, reassembly, and installation of the carbon-11 target (for a detailed step-by-step procedure, see Supplementary Data).

**Figure 8:**
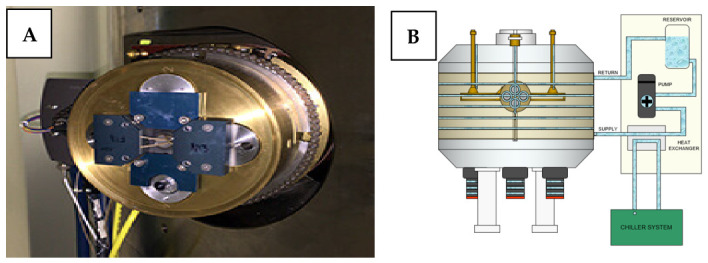
(**A**) Target changer and (**B**) cooling water system.

The target changer vacuum port is a critical component that facilitates target changes while maintaining the necessary high-vacuum environment for particle acceleration. This system features a rotating-style target changer capable of holding up to four ports (see Figure 8A). The target to be serviced is rotated to the service port position, cooling water is drained, and the target is inspected, with seals being replaced as needed (see Figure 8B). The vacuum isolation valve (three-way valve) is used to separate the target assembly from the service vacuum port and main cyclotron vacuum tank, allowing for target maintenance without venting the entire vacuum chamber.

The Siemens Eclipse HP cyclotron uses an automated target changer system designed for high-efficiency production of PET radioisotopes and beam strike targets. The target changer is located on two sides of the cyclotron, denoted as beamline 1 (BL-1) and beamline 2 (BL-2). Each target changer houses four targets with a gear-type motor and changer switch to allow forward and reverse rotation. A chain-driven mechanism turns the target barrel to allow for the servicing of a target or for positioning for beam bombardment. Some common target changer issues that can occur prior to production include failure to rotate to the requested position because the target changer and stationary parts are not perfectly aligned. Another issue is the uneven O-ring contact, which may occur when the O-ring presses against sharp metal corners during the transition to the target position and results in a tear in the face seal O-rings. This tear can cause a vacuum leak during rotation, triggering a sudden stop in the rotation or shutdown of the cyclotron. Misalignment of the changer switches may also prevent rotation to the desired position. If a target changer fails to rotate into position on either beamline, less activity is produced for daily production, resulting in the use of a single beamline instead of a dual target run.

**Figure 9:**
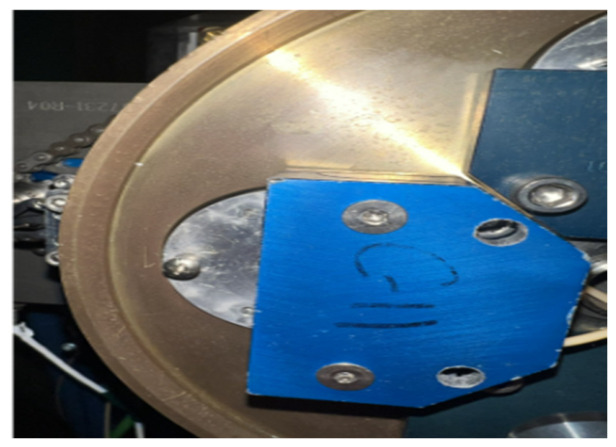
Umbilical assembly load and unload peek line connection.

Each target is connected via an umbilical assembly that manages all high-pressure seals, including the target window and product load and unload peek line connections (see Figure 9). A common issue during target removal is the presence of trace amounts of water inside the carbon-11 target body from coolant draining. As [^11^C]CO_2_ is produced via proton bombardment of N.O.S. gas, water contamination directly reduces radiochemical conversions and yields. Mechanical misalignment or pinhole leaks in the target body can allow coolant water to enter the target chamber. A method has been developed to remove water and evaluate the target after rebuilding (see Supplementary Data, Section S3).

**Figure 10:**
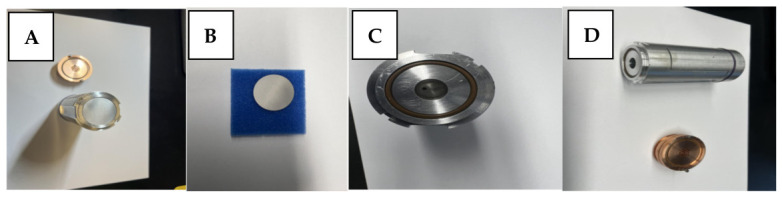
(**A**) Havar window in target, (**B**) Havar window, (**C**) target, and O-ring (**D**) target and copper nose piece.

Behind a lead-shielded L-block, the target is physically inspected, carefully wiped to remove any water around the target body, and then disassembled (see Figure 10A–D). The target copper nose piece is separated from the target body, and the Havar window is removed and inspected for discoloration (see Figure 10C,D). The Havar window is the most radioactive part of the target assembly and requires trained, experienced workers to handle it during target rebuilds or cleaning. The Havar window should be disposed of by placing it in long-term decay storage due to its high level of long-lived radiation. Rebuilding the target involves replacing the target window and installing new O-rings (see Figure 10D).

For an initial deployment of a new target, the nose piece and body are cleaned using ultrasonication (30 min per solvent) with the following chemicals ordered as follows: chloroform, acetone, methanol, HPLC-grade water, and deionized water. The aluminum target body is cleaned with this method only prior to its initial use in service, as a one-time process to remove manufacturing impurities or contaminants. Periodic baking of the nose piece to outgas contaminants has proven beneficial for maintaining low isotopologue mass. New seals and target Havar windows are wiped with methanol and prepared for installation (see Figure 10B,C).

For targets in current operation, solvent cleaning with organics is avoided unless the aluminum targets become discolored or corroded. Routine cleaning is accomplished with deionized water. In such unusual cases where an organic solvent is needed, the surface must be rinsed with deionized water several times, followed by drying with nitrogen gas before use.

Maintenance of the target is kept to a minimum, with the target left unaltered unless a failure to produce activity occurs [43]. If there are no problems with the target, preventive maintenance is carried out every six months. In a typical [^11^C]CO_2_ production setup, vacuum behavior during unloading serves as the primary diagnostic indicator: if the tank vacuum degrades significantly beyond the usual baseline, or fails to recover quickly after unloading, this is a reliable sign that the target must be rebuilt. Degrading O-rings or a ruptured Havar window will compromise the tight seal required to maintain the pressure differential during unloading. At that time, the Havar window and O-rings are changed; the target is inspected for internal build-up, and the color documented. During production the proton beam creates [^11^C]CO_2_ via ^14^N[p,α]^11^C reaction, causing oxidation to build up inside the target surface walls. Operators of the targets should continue to monitor during maintenance if the initial oxidation color has changed causing a lower yield. No attempts should be made to clean the internal surfaces while the target is still performing at acceptable value. In the event of target failure, surface cleaning and isolation checking are conducted. Only the Havar foil window needs to be changed as a form of preventive maintenance related to the carbon-11 target. All loading and unloading are monitored during runs, and any changes are reported to the radiochemistry group.

### 2.3. Target Conditioning and Switching Valves

**Figure 11:**
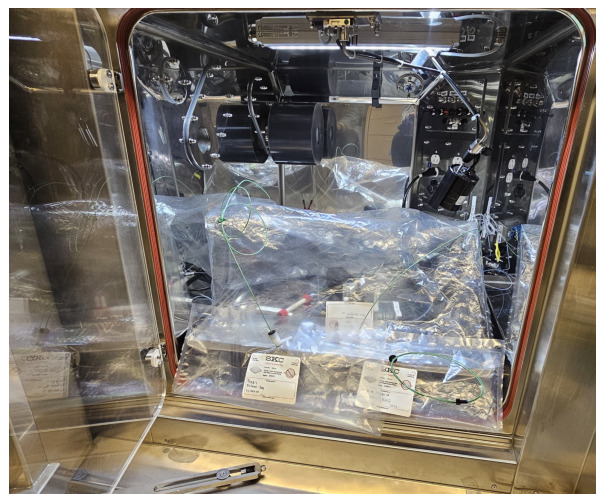
Exhaust bag in a hot cell shielded with a switching valve.

After reassembly and installation, the target must be conditioned to stabilize it before regular use. The goal of conditioning is to remove contaminants—particularly atmospheric nitrogen, moisture, and stable carbon—that could increase isotopologue mass and lower the molar activity of the final radiotracer. Conditioning involves high-vacuum evacuation followed by repeated flushing with N.O.S. gas, with the effluent directed to the exhaust bag in the hot cell (see Figure 11). All tanks directly associated with the carbon-11 target, namely the N.O.S. tank and the helium tank used for purging radioactive gas, remain open during this process. Previous issues have occurred when the N.O.S. tank was kept closed after the production run. The tank regulator developed a leak such that, when opened for production, it caused higher-than-normal isotopologue mass in the subsequent runs. The tank regulator was replaced and verified before placing back into production. Keeping the associated tanks open helps maintain carbon-12 at acceptable levels. By keeping these lines open during the process, any external carbon-12 is prevented from seeping into the closed system, thereby avoiding dilution of the radioactive carbon-11 produced in the target. The use of scrubbers on gas tanks is a standard practice to minimize impurities and traces of moisture from the tanks but is not necessary or implemented at this site.

Following gas flushing, the target is pressure-checked for 20 min at 290–330 psi, the optimal range for production loading. If the target pressure does not hold, or a notable change in tank vacuum is observed, the most common causes are misalignment of the nose piece to the target body, improper seating of the Havar window, or a pinhole leak. These issues must be resolved and the pressure test repeated before any irradiation testing is performed (for detailed troubleshooting procedures, see Supplementary Data, Section S3).

**Figure 12:**
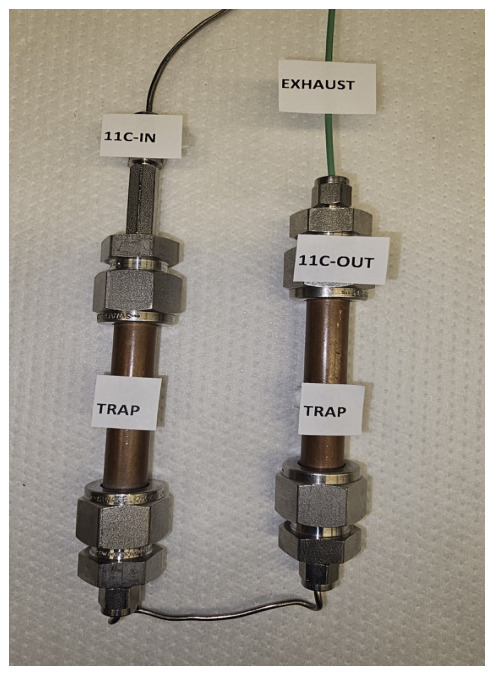
Molecular sieve trap.

Once the pressure test passes, select the switching valve flow direction from the target to the molecular sieve trap for testing (see Figure 12). The molecular sieve trap is a specialized device used to capture and concentrate radioactive [^11^C]CO_2_ produced by the cyclotron, and the captured activity is assayed with a dose calibrator. A graded conditioning bombardment is then performed to outgas the target, reduce isotopologue mass, and verify that the target can handle the thermal and radiation load. The target is irradiated at 50 µA for successive intervals of 5, 10, 20, and 40 min, with the cyclotron tank vacuum monitored throughout testing. Results are recorded in the daily logs and compared with previous values and daily production run data. All maintenance details, including the rebuild date and conditioning results, are documented and discussed at the weekly meetings. If target activity is below expectations, further troubleshooting and investigation is needed. It is not suggested to move forward on such a target if it does not meet the criteria and parameters stated from previous test runs.

All conditioning tests are performed using a single target. Once the target passes, a dual-run bombardment is used to verify the cyclotron’s ion source, tank vacuum, and pressures at dual target current. Both parties then communicate that the targets are ready for a production run.

**Figure 13:**
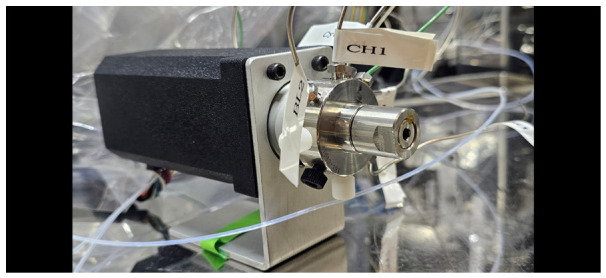
The VICI switching valve delivery system located in the hot cell.

Following bombardment, [^11^C]CO_2_ is delivered to the General Electric (GE) Fx2 C Pro automated module via a Valco Instruments (VICI) stainless steel 8-port switching delivery system (see Figure 13). Stainless steel lines are used for all [^11^C]CO_2_ transfer to minimize carbon-12 contamination, preserve high molar activity, and because their durability allows them to maintain integrity over time. A stainless-steel line is used from the N.O.S. tank regulator to the TSU for gas distribution to the carbon-11 target manifold. The 8-port switching valve receives [^11^C]CO_2_ from the cyclotron and enables delivery of target materials to multiple locations within the facility, providing the flexibility required for multi-target cyclotron configurations.

### 2.4. Automated Modules—Maintenance and Intrinsic Test Runs

The synthesis and conversion of the target gas, [^11^C]CO_2_, delivered from the cyclotron take place on GE TRACERlab Fx2 C Pro automated modules. Maintenance is required on these systems weekly, with consumables monitored daily (for a complete maintenance protocol and timeline, see Supplementary Data, Section S4).

**Figure 14:**
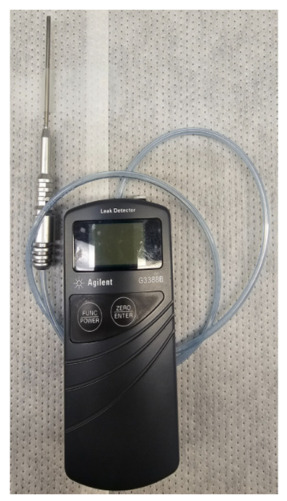
Leak detector.

**Figure 15:**
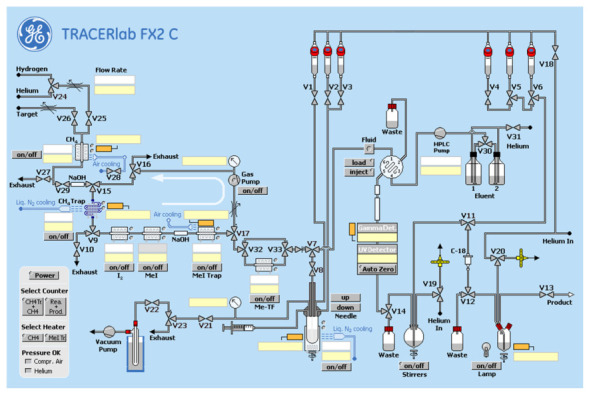
Graphical user interface for GE TRACERlab Fx2 C Pro automated synthesizer.

Leak integrity is verified prior to each test or production run using two complementary methods: an external Agilent helium leak detector (see Figure 14) and the module’s internal pressurization test, in which pathways are pressurized and closed, with the helium flow readout set at or close to zero (see Figure 15). The Agilent helium leak detector serves as an essential laboratory tool for detecting low-level leaks that the synthesis modules cannot detect. Consumable traps on the module are replaced on schedules determined by operational experience: the first iodine trap is changed every 10 to 12 runs, while the second iodine trap is changed monthly. The iodine chamber is measured at the beginning of each week, with iodine replenished monthly. The water trap (sicapent) is changed yearly but visually inspected and noted in the synthesizer replacement checklist at each use. The methyl iodide and methyl triflate traps are assessed through the production of [^11^C]CH_3_I and its conversion to products formed using [^11^C]CH_3_OTf (e.g., [^11^C]PiB); typical cleaning and replacement of these traps occur every two years. Tubing and PEEK lines are visually inspected before each production and replaced when frayed or weakened. The synthesizer replacement checklist is used to track all trap changes, with dates and operator inspections recorded for each component.

Following any maintenance on the automated module, a verification test run is performed before the system is returned to production. This test consists of an intrinsic run—conducted without the addition of cyclotron gases or external components—followed by a radioactive bombardment and conversion into [^11^C]CH_3_I. The intrinsic run establishes a baseline measurement of CH_3_I isotopologue mass originating solely from the module itself, independent of the cyclotron target and delivery lines. Typical intrinsic methyl iodide values observed on these modules are 5.43 ± 1.06 µg/mL (*n* = 5). This baseline is a critical diagnostic: if intrinsic isotopologue mass exceeds the expected range, the source of carbon-12 contamination can be attributed to the module rather than the cyclotron target or delivery system.

Once an acceptable intrinsic baseline is confirmed, the cyclotron is added to the system with a bombardment and delivery of [^11^C]CO_2_. Each beamline can be isolated independently, allowing one to determine whether a problem exists with a specific target. The conversion of [^11^C]CO_2_ to [^11^C]CH_3_I is determined, along with the isotopologue mass of CH_3_I. Typically, the addition of cyclotron gases to the automated module results in an approximately nine-fold increase in CH_3_I isotopologue mass compared with the intrinsic baseline. This increase reflects the contribution of carbon-12 from the target, gas purity, and delivery lines, and provides a direct measure of the cyclotron system’s contribution to total isotopologue mass. After each test run, an aliquot is removed from the reaction vessel and delivered to the quality control laboratory for analysis by radio-HPLC.

### 2.5. Carbon-11 Radiotracer Isotopologue Mass and Molar Activity

At NYUGSoM, carbon-11 radiotracers are produced for both human and animal studies. The Siemens Eclipse HP cyclotron produces the raw radionuclide, which is used to label these radiotracers in the form of [^11^C]CO_2_. This raw radioactivity is then delivered through a switching valve to an automated module, GE TRACERlab Fx2 C Pro, where it is converted into the electrophilic precursor, carbon-11 methyl iodide, or carbon-11 methyl triflate ([^11^C]CH_3_I or [^11^C]CH_3_OTf). Depending on the nucleophilic substrate and conditions set in the automated module, unique radiotracers are synthesized, purified, and formulated to provide the desired radiopharmaceuticals used for PET studies.

Table 1 summarizes laboratory-tracked data on overall activity produced, isotopologue mass (µg/mL), and molar activity (GBq/µmol) for each specific radiotracer. A typical conversion of cyclotron target gas, [^11^C]CO_2_, to [^11^C]CH_3_I on the GE TRACERlab Fx2 C Pro is approximately 33%. It should be noted that the radiotracers [^11^C]MRB and [^11^C]ER-176 are produced using a loop method with [^11^C]CH_3_I as the electrophilic precursor, whereas the synthesis for [^11^C]PiB and [^11^C]mHED requires further conversion of [^11^C]CH_3_I to [^11^C]CH_3_OTf as the precursor [23,44]. The synaptic density imaging agent, [^11^C]UCB-J, is formed using a traditional heterogeneous reaction vessel method by way of a palladium-coupled methylation ([^11^C]CH_3_I as the reagent), which requires activation of the boronic acid precursor for successful labeling [45].

### 2.6. Analytical HPLC for Determination of Isotopologue Mass

**Figure 16:**
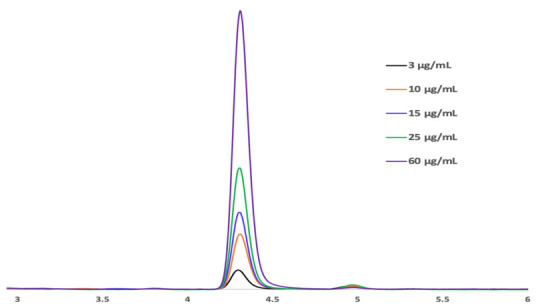
Representative HPLC chromatograms of methyl iodide calibration standards (3–60 µg/mL) showing a well-resolved methyl iodide peak at approximately 4.3 min on a C-18 column (mobile phase, 80:20 MeCN:H_2_O; flow rate, 1.0 mL/min; UV, 250 nm; column temperature, 40 °C).

A representative chromatogram for methyl iodide calibration standards spanning 3–60 µg/mL is shown in Figure 16. A well-resolved methyl iodide peak was observed at approximately 4.3 min under the selected chromatographic conditions, with peak area increasing proportionally with concentration across the calibration range. For each concentration level, five replicate injections were acquired, and the resulting peak areas demonstrated good repeatability in both magnitude and retention time over the course of the calibration sequence.

The method exhibited adequate sensitivity for the intended application, with a limit of quantification (LOQ) of 3.0 µg/mL and a limit of detection (LOD) of 0.9 µg/mL as derived from the calibration data. The resulting calibration curve (see Supplementary Data, Section S6) displayed a linear response over the 3–60 µg/mL range and fulfilled commonly accepted criteria for linearity and back-calculation accuracy across all standards. The validated calibration model was subsequently applied to determine the methyl iodide isotopologue mass in [^11^C]CH_3_I test runs. For [^11^C]CH_3_I, analytical radio-HPLC measurements were used to determine the mass of carbon-12 methyl iodide, as well as the radiochemical purity of carbon-11 methyl iodide for test runs and to track the performance of the automated module and carbon-11 target system.

The method employed to evaluate the performance of automated carbon-11 modules, as well as the condition of the cyclotron carbon-11 target and gases, relies on the use of analytical radio-HPLC. A five-point calibration curve using methyl iodide is generated to identify and quantify the amount of carbon-12 methyl iodide as well as to identify the radioactive [^11^C]CH_3_I (see Supplementary Data, Section S6). This, in turn, allows assessment of the current state of the automated module, delivery lines, and cyclotron target by recording and trending the molar activity. The linear calibration model over 3–60 µg/mL, combined with these performance characteristics, supports reliable back-calculation of methyl iodide mass in unknown samples and thereby underpins robust estimation of molar activity.

Application of this calibration to [^11^C]CH_3_I test runs allowed direct quantification of the intrinsic methyl iodide mass present at the time of analysis. For test runs, an intrinsic production run was first performed, followed by runs using cyclotron gas from bombardment of beamline 1 and then beamline 2, to provide data on module performance. This allows for baseline measurement from the intrinsic run, followed by an assessment of how external factors and different experimental configurations affect these values. In a representative production run, the average methyl iodide isotopologue mass was determined to be 44.32 ± 1.85 µg/mL (*n* = 3). Taken together, these results show that the radio-HPLC assay not only provides reliable identification and quantification of both [^11^C]CH_3_I and its isotopologue mass but also functions as a practical tool for ongoing surveillance of the cyclotron target system and automated synthesis modules. By trending the measured carbon-12 methyl iodide mass, and thus A_m_, across multiple [^11^C]CH_3_I batches, operators can identify gradual degradation of target, gas purity, or module performance and intervene before significant loss of molar activity or production reliability occurs. In this way, the analytical method serves as a critical link between routine quality control of [^11^C]CH_3_I and proactive maintenance for carbon-11 production infrastructure.

### 2.7. Radiotracer Release Criteria and Dose Administration

The quality control laboratory at NYUGSoM Radiochemistry Facility tests several specific parameters for each production batch prior to the release of a radiotracer. Quality control includes the following tests, not related to radio-high performance liquid chromatography (radio-HPLC): visualization of product appearance, verification of filter integrity, determination of radionuclidic identity, pH, strength, residual solvents, endotoxins, and sterility. Radio-HPLC, as part of the quality control process, is used to determine the radiochemical identity, radiochemical purity, and chemical purity, and plays a role in the determination of molar activity.

To ensure the prompt administration of the radiotracer, the clinical staff prepare the research participants prior to its release. At the NYUGSoM PETMR Center, the quality assurance (QA) team is in communication with the nuclear medicine technologists throughout the scheduled day of the exam and during the quality control process. Research participants need to be ready for injection at the time of radiotracer release to ensure that the radiotracer can be administered within the activity specifications for the study. The research participants need to have intravenous (IV) access in place before tracer delivery. Certain research studies also require additional testing procedures prior to radiotracer administration, all of which should be performed as early as possible so tracer delivery can be timed accordingly if there are delays in the participant’s preparation. Examples of additional participant testing requirements include vital-sign measurements or urine HCG testing [46]. The acquisition of the PETMR scan begins after an “uptake” period specified by the study design. If no uptake time of the radiotracer is required (e.g., dose administered while the patient or subject is on the camera or the study involves a dynamic acquisition) it is crucial to have all PET and MRI sequence plans acquired while the dose is being released and drawn for the study. Individualized doses are drawn for all research participants immediately prior to injection, as the final administered dose must be within specified study parameters. Research studies that utilize carbon-11 radiotracers need to be coordinated with the timing of the administered dose; this ultimately depends on communication among several teams—particularly the NYUGSoM radiochemistry team and the Siemens Healthineers cyclotron team—involved in radiotracer production.

### 2.8. Radioactive Stack Monitor Releases—Radiation Safety and NYSDEC Guidelines

During cyclotron production of PET isotopes, byproduct radioactive gases may be released into the atmosphere through the exhaust stack. Gas release activity can be minimized by utilizing trapping methods such as bagging, using filters (e.g., carbon or soda lime), or employing a decay tank [47,48,49].

U.S. Nuclear Regulatory Commission (NRC) regulation 10 CFR Part 20 states that “the individual member of the public likely to receive the highest dose will not be expected to receive a total effective dose equivalent in excess of 10 mRem (0.1 mSv) per year from these emissions”. Without proper gas trapping methods, it is challenging for a high-volume production facility to comply with these regulatory requirements to reduce radiation exposure to the public and staff [43].

### 2.9. Weekly Siemens Healthineers/NYUGSoM Cyclotron Alignment Meetings

A distinctive feature of carbon-11 production at NYUGSoM is that the cyclotron is operated and maintained by Siemens Healthineers, while the production of carbon-11 research doses is performed by the NYUGSoM Radiochemistry Group. These two organizations have different primary operational priorities: Siemens Healthineers’ commercial production is centered on fluorine-18 radiopharmaceuticals, whereas NYUGSoM’s carbon-11 program requires consistently low carbon-12 contamination and high molar activity. This difference in focus means that carbon-11 target condition, which is critical to the NYUGSoM program, may not be a routine priority for the cyclotron operations team. Without a formal communication structure, gradual degradation in target performance, vacuum behavior, or ion source condition could go unrecognized until production failures occur.

To address this, the two teams hold a weekly alignment meeting every Thursday, typically lasting 30 min, with a structured agenda. Discussion topics include cyclotron target performance from the preceding week, any observed changes in vacuum behavior during loading and unloading, ion source usage and current parameters, upcoming preventative maintenance windows, molar activity trends across recent production runs, and any anomalies or incidents requiring investigation. The meetings also serve as the forum for coordinating the maintenance activities described in Section 2.1, Section 2.2, Section 2.3 and Section 2.4—scheduling target rebuilds, planning conditioning bombardments after maintenance, and reviewing intrinsic test run results before returning systems to production.

This structured communication has proven essential for maintaining carbon-11 production quality. The meetings provide the mechanism through which both teams jointly monitor ion source wear (Section 2.1), coordinate target cleaning and conditioning schedules (Section 2.2 and Section 2.3), and review automated module performance data (Section 2.4). By establishing a recurring, agenda-driven forum with open participation from both the university and industry teams, operational problems are identified and addressed proactively rather than reactively.

## 3. Materials and Methods

### 3.1. Chemicals and Reagents

All reagents and starting materials were purchased from commercial suppliers and used without further purification, unless otherwise noted. A sodium iodide detector (Eckert & Ziegler, Robert-Rössle-Straße 10 13125 Berlin, Germany) coupled in series with a photodiode array detector (Shimadzu SPD-20MA, Shimadzu Scientific Instruments 7102 Riverwood Drive, Columbia, MD, USA, UV/Vis set at λ = 250 nm) was used for the identification of radiolabeled [^11^C]CH_3_I. Data acquisition for analytical systems was accomplished using LabSolutions LC software (Version 6.129, Shimadzu Scientific Instruments 7102 Riverwood Drive, Columbia, MD, USA). [^11^C]Carbon dioxide was produced on a Siemens Eclipse HP cyclotron (11 MeV). Methyl iodide (certified reference material, 99.9% purity, Sigma-Aldrich, St. Louis, MO, USA) was used as the reference standard. Research-grade helium, hydrogen, compressed air, and ultra-high purity N.O.S. gas (oxygen: 1.013%, nitrogen: balance, Part No. X02NI99C15A3091) were purchased from Airgas US LLC (Cherry Hill, NJ, USA). A leak detector (Agilent Technologies, Inc. 2850 Centerville Road Wilmington, DE, USA, Part No. G3388B) was used for leak checks with the use of helium gas. Iodine, resublimed crystals (Fisher Scientific L.L.C. US, 1 Reagent Lane Fair Lawn, NJ, USA; Country of Origin: Japan, Part No. I35-100), phosphorus pentoxide (Sigma-Aldrich, 3050 Spruce Street, Saint Louis, MO, USA; Quality Assurance Origin: Buchs, Switzerland, Part No. 79610-100G), and nickel catalyst (Shimwa Chemical Industries LTD, 50-2 Kagekatsuch Fushimiku, Kyoto, Japan, Part No. 66062) were stored in a desiccator. Acetonitrile, ascarite II, porapak Q, and carboxen adsorbent were purchased from Sigma-Aldrich (St. Louis, MO, USA). Deionized water (Merck/EMD Millipore Frankfurter Strasse 250, 64293 Darmstadt, Germany) was generated and used in-house. A benchtop scale (120 g capacity, 0.1 mg readability; Mettler Toledo, Columbus, OH, USA) was used to weigh chemicals. 

### 3.2. Cyclotron Bombardment

Prior to each production bombardment, the radiochemistry team and Siemens Healthineers staff communicate regarding the preconditioning requirements and the type of bombardment needed for the day. Each beamline target is manually conditioned through a series of preparatory steps. The target is charged with N.O.S. gas to a pressure of 310 psi (beamline 1) and 320 psi (beamline 2), after which the pressure is released into a waste bag. This pressurization and release cycle is repeated three times per beamline to purge any residual contaminants from the target chamber. Following this conditioning, each beamline undergoes a series of carbon-11 target bombardment conditioning runs, in which the cyclotron initiates bombardment at 45 μA for 2 min, and the resulting activity is released directly into the waste bag. This process is performed up to three times per beamline, bombarding and unloading one beamline at a time, to further condition the target and ensure consistent performance before production begins. Production bombardment for [^11^C]CO_2_ is then carried out using either a single beam at 55 μA or dual beam at 45 μA for a duration determined by the NYUGSoM radiochemistry production team. Prior to completion of bombardment, Siemens Healthineers staff members call the radiochemistry group to confirm they are ready to receive; if the radiochemistry team is not ready to receive [^11^C]CO_2_, additional time is added to the cyclotron’s bombardment. Once radiochemistry is ready and their switching valve is designated for the correct synthesis unit, the activity is unloaded and delivered directly to the synthesis module. This is followed by a helium sweep to ensure complete transfer of activity. Siemens Healthineers staff members and the NYUGSoM radiochemistry team are always in contact throughout the process. Stack monitoring of the daily release is viewed during the bombardment and radiosynthesis.

### 3.3. Radiochemistry

Two hours before receiving carbon-11 for a production run, a leak check was performed on the synthesis module with the use of helium gas and a leak detector. GE TRACERlab Fx2 C Pro synthesis modules are used for all carbon-11 productions with an embedded time list. Prior to receiving radioactivity from the cyclotron, the ovens associated with the automated module are heated and conditioned in the following order and for the designated times: methane oven: 100 mL/min of hydrogen gas heated to 350 °C for 20 min; methane trap: 50 mL/min helium gas heated at 120 °C for 10 min; methyl iodide trap: 50 mL/min helium heated to 190 °C for 10 min (see Figure 15). Following conditioning, a test without radioactive material is performed to verify that the time sequence and all heating and cooling systems are operational prior to daily production. The synthesis unit is then conditioned and ready for daily production.

An in-house generated and validated automated time list is uploaded from the Fx2 C Pro module and executed 20 min before the unload of target gas ([^11^C]CO_2_) from the cyclotron. For intrinsic runs, the procedure follows the method outlined below without the use of the cyclotron or any gases associated with the cyclotron and its delivery. During this automated process, the methane oven is heated to 350 °C for 2 min, followed by its cooling to <45 °C. Once the desired temperature of the methane oven is reached, the cyclotron target is unloaded into the automated module. The operator must accept the gas to initiate the time sequence. The following sequence is executed from the built-in time list: target gas (either bombarded N.O.S. gas to produce [^11^C]CO_2_ via ^14^N[p,α]^11^C nuclear reaction or unreacted N.O.S. gas for baseline runs), is unloaded into the automated module and directed into the methane oven. Radioactive carbon dioxide gas is trapped in the methane oven, and the system is filled with hydrogen gas and sealed. The oven is then heated to 350 °C in the presence of hydrogen gas and filled with a nickel-shimalite catalyst, which, when heated under hydrogen gas, converts the carbon dioxide gas into methane ([^11^C]CH_4_) gas. Both the unconverted [^11^C]CO_2_ and the formed water from the hydrogenation process are trapped by an ascarite column (sodium hydroxide) and phosphorus pentoxide column (Sicapent). The [^11^C]CH_4_ produced is then released from the methane oven and directed to the methane trap, which is filled with carboxen adsorbent and cooled to −75 °C in the presence of liquid nitrogen for further purification and concentration. The [^11^C]CH_4_ is then converted to methyl iodide ([^11^C]CH_3_I) by passing it through iodine vapor, sublimed at 720 °C, via an internal gas circulating system, with CH_4_ trap heating at 80 °C. The formed hydrogen iodide (HI) is trapped by a separate ascarite trap. The formed [^11^C]CH_3_I is separated from this circulation process using an inline trap containing porapak Q as an adsorbent. The unconverted [^11^C]CH_4_ is allowed to recirculate in the presence of iodine at 720 °C. At the end of the circulation process, the collected [^11^C]CH_3_I is heated off the adsorbent (190 °C) and directed into the reactor with the use of helium at 15 mL/min. For the test runs, [^11^C]CH_3_I is collected in the reactor at −20 °C until the maximum activity is reached. The automated process is then terminated, and a sample is removed for radio-HPLC analysis.

### 3.4. Radio-HPLC Analysis

HPLC was used to establish and validate a calibration curve for the quantification of methyl iodide mass, with methyl iodide at known concentrations serving as the primary standard. Using a 1000 µL calibrated micropipette, 1.0 mL of neat methyl iodide was transferred into a tared 100 mL volumetric flask placed on a weighing balance, and the corresponding mass was recorded. The flask was then filled to volume with a diluent consisting of 80% acetonitrile and 20% deionized water to obtain a primary stock solution (25 mg/mL). An intermediate stock solution (500 µg/mL) was prepared by dilution of the primary stock with the same diluent.

Calibration standards were prepared in acetonitrile: water (80:20, *v*/*v*) at five nominal concentrations spanning 3–60 µg/mL by dilution of the intermediate stock using a calibrated pipette. All standards were prepared in 7 mL vials and mixed thoroughly. Fresh calibration solutions were prepared on the day of analysis and stored in sealed vials to minimize volatilization losses.

Chromatographic analysis was performed on a C_18_ reversed-phase column (Luna C18(2), 250 × 4.6 mm, 5 µm, 100 Å) thermostatted at 40 °C, using an isocratic mobile phase of acetonitrile: water (80:20, *v*/*v*) at a flow rate of 1.0 mL/min. UV detection was carried out at 250 nm with an injection volume of 20 µL, and the total run time was 10 min.

System suitability was assessed at the start of each analytical sequence using a mid-range calibration standard by monitoring signal-to-noise ratio, peak tailing factor, and retention factor, as well as retention time and peak area repeatability. In addition, uracil was injected to determine the column void volume [50].

For each calibration level, five replicate injections were acquired, and the mean iodomethane peak area was plotted versus nominal concentration. Linear regression was applied to construct the calibration curve using LabSolutions LC software. Linearity was evaluated based on the coefficient of determination (R^2^) and the agreement between back-calculated and nominal concentrations across the calibration range (see Supplementary Data). Limits of detection and quantification, accuracy, and precision were assessed in accordance with ICH Q2(R2) guidelines, and the validated calibration model was subsequently used to quantify the carbon-12 methyl iodide mass in [^11^C]CH_3_I test batches. As [^11^C]CH_3_I serves as a precursor in various synthetic pathways for our carbon-11 radiolabeled tracers, its radiochemical identity and purity were also determined using radio-HPLC equipped with a photomultiplier tube (NaI) radioactivity detector.

## 4. Conclusions

The window for a successful carbon-11 radiotracer injection is tight, with a 1 h expiry time for most carbon-11 radioligands produced at our facility. More than a quarter of this time (25 min) is used for quality control testing and release of the radiopharmaceutical, leaving just 35–40 min for delivery to the clinic and administration to the participant. This timeline demands that the cyclotron, radiochemistry production, quality control, and clinical teams operate in close coordination from the start of each production day.

This manuscript describes how the NYUGSoM Radiochemistry Facility and Siemens Healthineers have developed an integrated operational framework for maintaining consistent, high-quality carbon-11 production within an academic–industry partnership. The key elements of this framework include (1) cyclotron target maintenance and conditioning protocols that minimize carbon-12 contamination of [^11^C]CO_2_; (2) automated module maintenance schedules that maintain low intrinsic isotopologue mass; (3) a validated radio-HPLC analytical method for trending methyl iodide isotopologue mass as a diagnostic tool for proactive maintenance; and (4) structured weekly alignment meetings and real-time communication between the university and industry teams.

The production data presented in Table 1 demonstrate that these integrated protocols consistently yield molar activities of 171–293 GBq/µmol across five carbon-11 radiotracers, well above the recommended minimum of 40 GBq/µmol for reversibly binding the radiotracers used in human PET studies [32]. Intrinsic test runs on the automated modules established a baseline isotopologue mass of 5.43 ± 1.06 µg/mL (*n* = 5), providing a reference point for attributing carbon-12 contamination to the module versus the cyclotron system.

Carbon-11 production sites face challenges in achieving and maintaining adequate molar activity, in part because cyclotron operators may be primarily trained on fluorine-18 production, where isotopologue mass is not a significant concern. This manuscript is intended to serve as a practical operational resource for radiochemists, cyclotron engineers, and facility managers working to establish or improve carbon-11 programs. We encourage other sites to publish their own operational approaches and benchmarks to build a shared knowledge base for the PET radiochemistry community.

## Figures and Tables

**Table 1:** Activity, isotopologue mass, and molar activity of carbon-11 radiotracers at EOS.

Compound	Average Decay-Corrected rcy (%)	Activity (GBq)	Mass (μg/mL)	A_m_ (GBq/μmol)
[^11^C]PiB (*n* = 141)	12%	3.4 ± 0.8	0.6 ± 0.51	171 ± 68
[^11^C]mHED (*n* = 8)	20%	5.5 ± 1.3	0.40 ± 0.10	293 ± 48
[^11^C]MRB (*n* = 70)	12%	3.0 ± 1.2	0.52 ± 0.24	190 ± 50
[^11^C]UCB-J (*n* = 6)	12%	2.6 ± 0.8	0.49 ± 0.30	230 ± 145
[^11^C]ER-176 (*n* = 25)	21%	5.4 ± 2.2	1.1 ± 0.5	194 ± 66

## Data Availability

Data are available from the corresponding author upon request.

## Supplementary Materials

The following supporting information can be downloaded at https://www.mdpi.com/article/10.3390/molecules31122095/s1. Figure S1. (A) Diffusion pump, (B) vacuum controller, (C) ion gauge filament, and (D) heater element; Figure S2: Ion source upper and lower rods, cathodes, collimators, and anode; Figure S3: Carbon 11 nose piece and target body; Figure S4: [^11^C]CO_2_ output verses beam time for beamline 1 (BL 1) and beamline 2 (BL 2). Note, all data derive from a single bombardment at a current of 50 μA.; Figure S5: Calibration curve for methyl iodide; Table S1: Items and maintenance schedule for the GE TRACERlab Fx2 C Pro. Refs. [51,52] are cited in the Supplementary Materials.

## Abbreviations

The following abbreviations are used in this manuscript:

[^11^C]ER-176	[^11^C]-(R)-*N*-sec-butyl-4-(2-chlorophenyl)-*N*-methylquinazoline-2-carboxamide
[^11^C]mHED	[^11^C]-meta-hydroxyephedrine
[^11^C]MRB	(*S,S*)-[^11^C]methylreboxetine
[^11^C]PiB	2-[4-[(^11^C)methylamino]phenyl]-1,3-benzothiazol-6-ol
[^11^C]UCB-J	(*4R*)-1-[[3-(^11^C)methylpyridin-4-yl]methyl]-4-(3,4,5-trifluorophenyl)pyrrolidin-2-one
[^11^C]CO_2_	Carbon-11 carbon dioxide
[^11^C]MeI	Carbon-11 methyl iodide
B*_max_*	Total receptor concentration
*BP*	Binding potential
Bq	Becquerels
Ci	Curies
CT	Computed tomography
EOB	End of bombardment
EOS	End of synthesis
GBq	Giga becquerels
GUI	Graphical user interface
H^-^	Hydrogen ion
HVAC	Heating, ventilation, and air-conditioning
K*_D_*	Equilibrium dissociation constant
LIS	Lab impex system
LOD	Limit of detection
LOQ	Limit of quantification
MeI	Methyl iodide
MeOTf	Methyl triflate
MeV	Mega electron volts
min	Minutes
mSv	Millisievert
NOAEL	No observed adverse effect level
NRC	Nuclear Regulatory Commission
μA	Micro amps
μmol	Micro moles
mrem	Milli roentgen equivalent man
MR	Magnetic resonance
A_M_	Molar activity
NYUGSoM	New York University Grossman School of Medicine
N.O.S.	1% oxygen in 99% nitrogen, specialty gas
PET	Positron emission tomography
psi	Pounds per square inch
H^+^	Proton
QA	Quality assurance
QC	Quality control
rcp	Radiochemical purity
rcy	Radiochemical yield
RF	Radio frequency
t_R_	Retention time
TOI	Time of injection
TSU	Target support unit
VICI	Valco Instruments
V_T_	Volume of distribution
*v*/*v*	Volume per volume
λ	Wavelength
