# Supplementary material for: Carbon-11 Production: Communication, Operations, Maintenance, Troubleshooting, and Analysis for Maintaining High-Grade Bombardment and Provisions of [11C]Carbon Dioxide and Its Conversion to [11C]Methyl Iodide
Source: Molecules. 2026 Jun 15;31(12):2095. doi: 10.3390/molecules31122095 (PMC13304833; doi:10.3390/molecules31122095)
Supplement: Supplementary file 1 [file molecules-31-02095-s001.zip › molecules-4315303-supplementary.pdf]

molecules

Supplementary Materials

Carbon-11 Production: Communication, Operations, Maintenance, Troubleshooting, and Analysis for Maintaining High-Grade Bombardment and Provisions of [^{11}C]Carbon Dioxide and Its Conversion to [^{11}C]Methyl Iodide

Simon K. Joseph [1], Andrew Tavare [2], Kiara Thomas [1], Dae-In Kim [3], Kaleigh Timmins [1], Melchor V. Cantorias [1], Briana Roman [1], Jakub Mroz [1], Jairo Baquero [1], Julian Calderin [2], Lucas Fernandez [2], Sandy Phung [2], Andrew Chung [2] and Patrick Carberry [1,*]

1 Department of Radiology, New York University Grossman School of Medicine, 660 First Avenue, Room 240, New York, NY 10016, USA; simon.joseph@nyulangone.org (S.J.)
2 Siemens Healthineers (PETNET SOLUTIONS NYU) 660 First Avenue, Room 140, New York, NY 10016, USA
3 Radiation Safety, Real Estate Development and Facilities, NYU Langone Health, Berkley Park, 339 E. 28th St, New York, NY 10016
* Correspondence: patrick.carberry@nyulangone.org (P.C.); Tel.: +1-646-501-9607

Table of Contents

1. Cyclotron Systems 2

2. Ion Source Preventive Maintenance 2

3. Carbon-11 Target Maintenance 3

4. Automated Module Maintenance 5

5. GE TRACERlab Fx2 C Pro Cleaning 7

6. Construction of Methyl Iodide Calibration Curve by HPLC 8

7. References 9

1. Cyclotron Systems

Numerous aspects of routine operation have direct implications for [^{11}C]CO_2 production quality and reliability. The cyclotron vacuum system is maintained by four oil-based heated diffusion pumps with butterfly valves (see **Figure S1A**). The vacuum system is controlled by a vacuum controller in the electrical cabinet (see **Figure S1B**). The offline vacuum of the tank is 9×10^{-08} Torr, and the initiated cyclotron vacuum is 5×10^{-06} Torr once hydrogen gas is allowed to flow into the cyclotron vacuum chamber for hydrogen ion production. The vacuum pressure is measured using an ion gauge dual filament present inside the vacuum chamber (see **Figure S1C**). If a target is blown or a major change in pressure occurs in the vacuum tank, one of the ion gauge filaments will burn out. If the primary filament burns out, the system can be switched to the secondary. A typical problem with the vacuum system occurs when diffusion pump heater elements burn out due to excessive heating conditions (see **Figure S1D**), with heater element failures indicated on the vacuum controller. Heater elements are kept in stock for replacement when the problem occurs. The heating elements often lack visible signs of failure, such as cracks, discoloration, or a burnt smell. A failed heating element will automatically shut down the cyclotron due to vacuum levels out of range. When internal components degrade without external damage, the most reliable way to confirm a problem is to test all four diffusion pump heater elements for electrical continuity using a multimeter or clamp amp meter. Vacuum problems are critical and create a slow recovery time for cyclotron production.

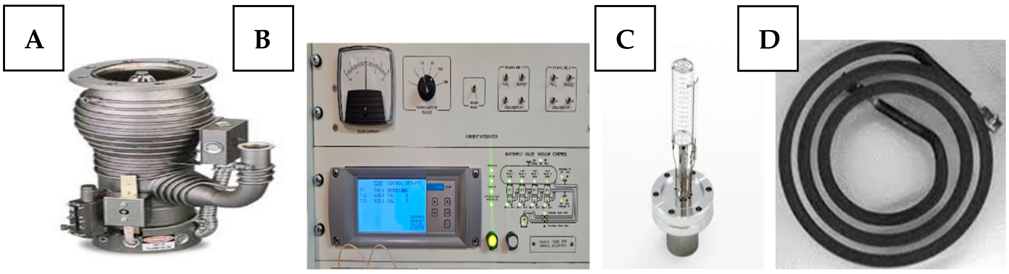

Figure S1. (A) Diffusion pump, (B) vacuum controller, (C) ion gauge filament, and (D) heater element.

2. Ion Source Preventive Maintenance

The Siemens Eclipse HP cyclotron requires PM that is designed to optimize ion source lifetime, reduce tuning time, optimize beam current and extend cathode/anode lifetime. Ion source rebuilds parts include two collimators, two cathodes, an anode, lower/upper rods, the O-ring of the upper rod, and upper/lower shafts. Both collimators are cleaned with isopropanol and light sandpaper, clearing the first coat for a shining mirror quality. Cathodes are visually inspected for threads and striking areas. Anodes are cleaned with sandpaper or sand blaster across their only inner area. Lower/upper rods are visually inspected. The upper rod O-ring is replaced with every cleaning. During disassembly, the upper rod's O-ring is a potential source of hydrogen leak from the ion source and may cause current drifting of ion source current during production. The upper/lower shaft can be cleaned with low sandpaper or by a sand blaster. All parts are placed in a beaker and sonicated with chloroform, acetone, and methanol for 30 min. The sonicated parts are dried with argon gas. Collimators, cathodes, and the anode are wrapped in aluminum foil, and baked in the oven at 260 °C for 10 min. The screws inside the central copper cooling housing are inspected. All parts of the ion source are reassembled; once rebuilt, current tuning and conditioning of the components are conducted (see **Figure S2**).

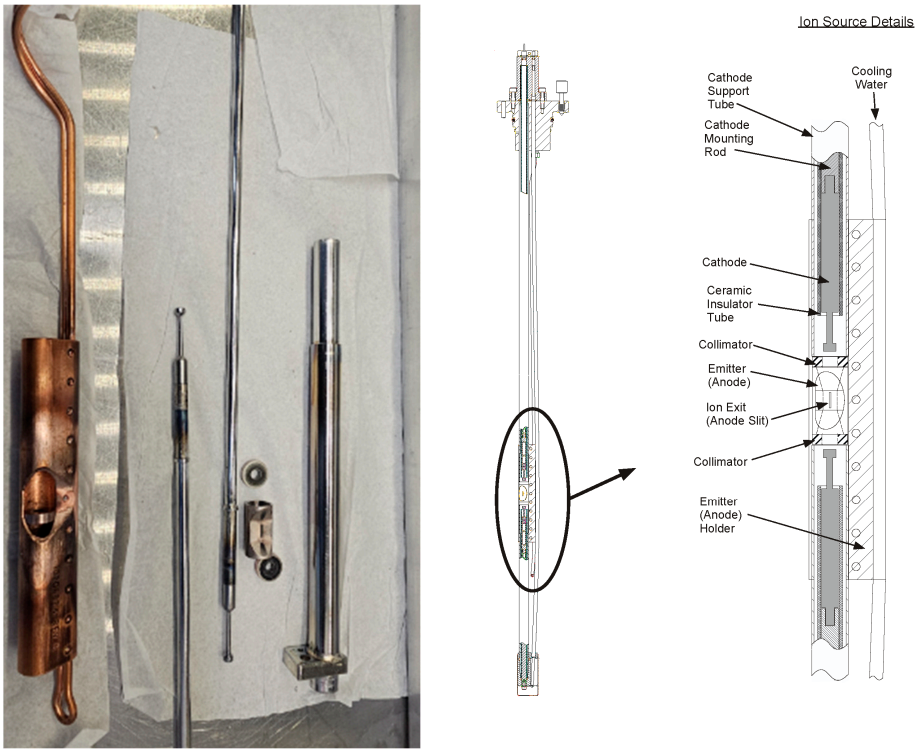

Figure S2. Ion source upper and lower rods, cathodes, collimators, and anode.

3. Carbon-11 Target Maintenance

The Siemens Eclipse HP 11 MeV cyclotron uses an aluminum-type target. Maintenance for carbon-11 cyclotron targets focuses on ensuring high radiochemical yield and minimizing radiation exposure to personnel. Due to the high-energy proton bombardment, the required standard maintenance is typically performed on a quarterly (6-month) basis. A new, unused carbon-11 target is to be handled differently from one that has been in routine production. For a new target, components are cleaned using an ultrasonic bath with solvents for 30 min each in the following order: chloroform, acetone, methanol, HPLC-grade water, and deionized water. Final rinsing must be used with deionized water, followed by drying with nitrogen gas or the target gas. The target is then wrapped in aluminum foil and placed in an oven at 260 °C for 10 min. The thin foil Havar window that separates the target gas from the cyclotron vacuum must be inspected for signs of thinning or rupture. The Havar window is cleaned with methanol, wrapped in aluminum foil, and placed in the oven at 260 °C for 10 min.

If any trace of water is observed when removing the target from the target changers, wipe the outer target body and copper nose piece area with a Kimwipe to remove water. Remove the nose piece, Havar window, and O-rings and store them safely. Place the remaining target body on a stand near the target changer. Reconnect the umbilical with the input and output single peek line connected to the target. Place a Kimwipe in the nose piece area, turn on the target gas, and dry the beam striking area for 15 min. This allows for all moisture and contaminants to be pushed out of the inner target body. Inspect the beam strike area to verify complete water removal. Rebuild the target using the kit and perform purges and test bombardment runs as usual. This method effectively clears all water and restores the target to its optimal gas composition and operating conditions.

Leak testing is critical because targets operate at high pressures over 900 psi during irradiation. The carbon-11 target consists of two sections - the nose piece and the target body (see **Figure S3**). The main body is made from aluminum while the nose piece is a copper hex-grid. The inner diameter of the carbon-11 target holds the starting material that consists of mixed gas, with the composition of 99% nitrogen and 1% oxygen (N.O.S.). The dimensions of the aluminum body are 10 mm by 90 mm in cylinder length. The Havar window allows the beam to strike the

filled target of N.O.S. gas at a starting pressure of 320 psi. During bombardment, the automated operating system will increase the pressure of the irradiated target to ~900 psi. There is one main inlet/outlet peek line of the target in which the flow direction comes from the valve peek manifolds associated with the target support unit apparatus. Following gas flushing, the target is pressure-checked for 20 min at 290–330 psi, the optimal range for production loading. If the target pressure does not hold, or a notable change in tank vacuum is observed, the most common causes are misalignment of the nose piece to the target body. Ensure the nose piece and target body connection section are aligned properly on all sides. Improper seating of the Havar window or O-rings misalignment, as well as a missing O-ring, will cause a leak. Check and verify that the O-ring is not torn. Pressure check the nose piece honeycomb area for a pinhole leak to the Havar window. If the leak check does not pass, replace it with a clean nose piece. These issues must be resolved, and the pressure test is repeated before any irradiation testing is performed. Once the test passes, set up the molecular sieve trap prior to any $[^{11}C]CO_2$ transferal to the synthesis unit. This step is used to verify the production of the target activity produced after cleaning and rebuilding. This also helps to further condition the target to lower contamination. For reference, $[^{11}C]CO_2$ produced for each beamline, beamline 1 and beamline 2, can be found in **Figure S4**.

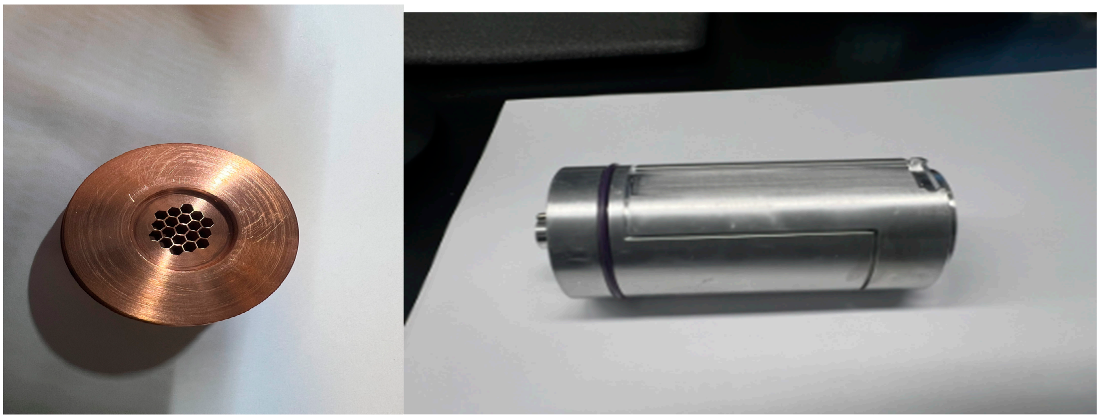

Figure S3. Carbon 11 nose piece and target body.

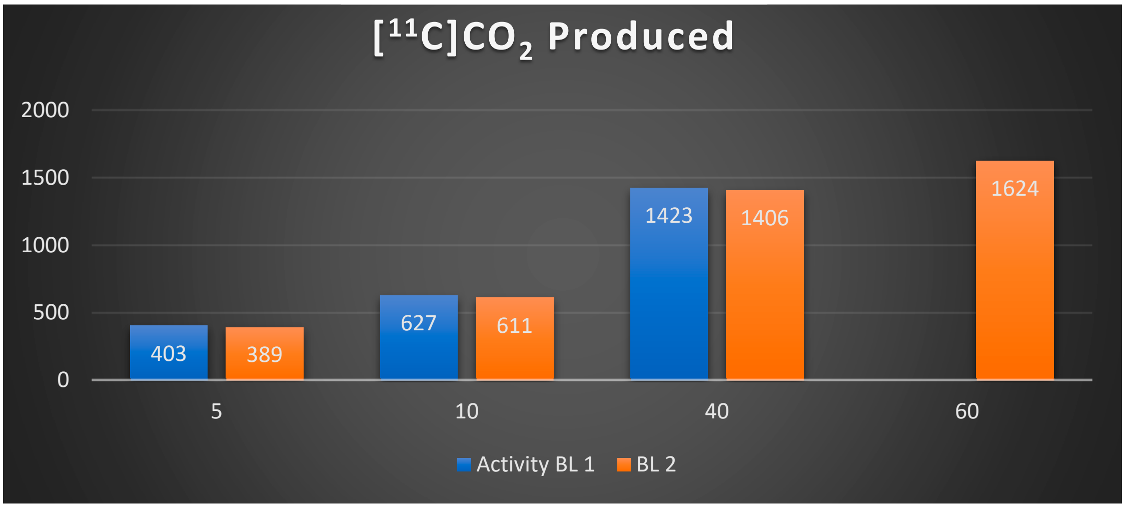

Figure S4. $[^{11}C]CO_2$ output verses beam time for beamline 1 (BL 1) and beamline 2 (BL 2). Note, all data are derived from a single bombardment at current of 50 μA.

4. Automated Module Maintenance

To ensure the safe and reliable operation of the GE TRACERlab Fx2 C Pro, maintenance is performed at specified intervals. The GE TRACERlab Fx2 C Pro manual provides detailed steps and a maintenance schedule to follow [1,2]. However, it is advisable to determine which maintenance tasks best support your site based on the type of radiosynthesis for each compound. Some maintenance tasks may not need immediate attention and should be scheduled accordingly. This table is in conjunction with yearly preventive maintenance provided by General Electric.

The vacuum pump is integrated with the synthesis unit to perform critical functions such as evaporating solvents and aiding in the transfer of reagents during synthesis, it is also used in the cleaning sequence to dry the reaction vessel and lines. The vacuum is controlled by GE software and programmed into various automatic timing sequences. The glass cold trap, lines, and connections are inspected daily. The cold trap is removed and cleaned with acetone and dried. The Dewar flask that houses the cold trap is filled halfway with liquid nitrogen before each automated sequence.

The exhaust system handles potential volatile radioactive gases produced during radiosynthesis. Radioactive exhaust gas can be collected in a gas bag or diverted into a decay tank for storage and eventual release. All internal systems that are vented are connected by valves or four-way connectors to the exhaust line through a soda lime trap inline to the exhaust bag. The exhaust line (1/8″) diameter is larger than the designated synthesis lines (1/16″) used in the module for operations and radiolabeling. The internal exhaust line is cleaned yearly with the use of deionized water, acetone, and acetonitrile and is dried for 20 minutes. Cleaning the exhaust helps with synthesis yield by clearing any cross-contamination that may be introduced during heating and cooling into the reaction vessel.

The reactor vessel is cleaned after every production run. From yearly preventive maintenance (performed by GE), the four lines plumbed into the reaction vessel (three inlets and one exhaust) are evaluated. When reagent or solvent delivery lines are too long or poorly designed, reagents may not reach the bottom of the reactor vessel, instead splashing onto the reactor walls, top, or directly into the exhaust pathway. This causes material to accumulate in the reactor headspace rather than mixing with the reaction mixture. If unreacted materials reach the exhaust system, this can lead to clogging of the exhaust line, increasing system pressure. This will also have a direct impact on the overall radioactivity in the synthesis, which may lead to low yields. All three inlet lines to the reactor vessel should be set and cut no longer than the reactor upper neck of the glass reactor vial. The transfer line should be set as the longest line when the dipper is raised; when lowered, the line should reach the bottom of the V-neck in the reactor vessel.

Proper maintenance of the vacuum pump, exhaust cleaning, and optimizing reactor line lengths are critical factors in enhancing overall yields and operational efficiency. Clean exhaust systems prevent pressure fluctuations that restrict flow, while optimized line lengths directly influence conversion times, synthesis time, and the reduction of unwanted competing reactions.

Table S1. Items and maintenance schedule for the GE TRACERlab Fx2 C Pro.

Item	Scheduled Maintenance	Operator Test	Maintenance Procedure
Septa	As needed	Visual inspection, leak check.	Replace septa as needed.
Teflon tubing	Yearly/as needed	Visual inspection, check for cracks.	Replace Teflon tubing when lines become frayed or cracked. Tubing associated with exposure to high radiation is changed annually.

Tank check (helium and hydrogen gas)	Change ≤ 500 psi	Monitor primary/secondary gauges.	Replace tanks as needed.
Reaction vessel	Cleaned after each run	Check seal. Clean reaction vessel.	Replace if damaged. Replace O-rings if not sealing properly.
Reaction vessel peek needle	As needed/yearly	Check and readjust the peek before each clean sequence.	Adjustments or cutting a new peek line may be required. Yearly replace the peek needle.
Valves	Daily	Exercise valves when performing manual pre-flushes. Check flow rates and observe blockage.	Replace valves as needed. Note: system has two-way and three-way valves.
Vacuum pump	Daily	Check vacuum pull; clean cold finger trap for each run. Fill Dewar with liquid nitrogen.	Fill the Dewar with liquid nitrogen before all runs. Clean out the cold finger trap before each run. If necessary, clean out the membrane of vacuum pump. For harsh conditions, clean vacuum lines with 1:1 acetone: deionized water solution.
HPLC pump	As needed	Purge system. Perform flow check.	Replace lines and tubing as needed. Sonicate valve if blockage/slow flow rate observed.
Fluid detector	Daily	Observe fluid detector responds during the cleaning sequence.	Adjustment of the screw in fluid detector may be necessary; if unresponsive, replace the fluid detector.
1st Iodine trap	Weekly	Visual inspection of color moving out of the trap (purple/brown).	Remove used ascarite, clean trap and tubing with water, acetone, and acetonitrile, dry under a stream of nitrogen for 5 min.
2nd Iodine trap	Monthly	Visual inspection of color moving out of the trap (purple/brown).	Remove used ascarite, clean trap and tubing with water, acetone, and acetonitrile, dry under a stream of nitrogen for 5 min.
Iodine (quartz glass)	3 months	Check the level of iodine in the quartz glassware weekly.	Add more iodine flakes to glassware, careful not to overfill. If blockage, clean it out with acetone and dry thoroughly.
Methyl iodide trap	2 years	Check for conversion of $[^{11}C]CO_2$ into $[^{11}C]CH_3I$; acceptance rate ≥ 30% conversion factor.	Clean and dry column, replace with glass wool and porapak Q, 50-80 mesh (0.40 g).

Methyl triflate trap	2 years	If rate of conversion drops for radiotracers that use [^{11}C]CH_3OTf in synthesis while the [^{11}C]CH_3I conversion is deemed acceptable.	Clean and dry the column, replace silver wool and silver triflate mixture (~0.9 mg).
Water trap	Yearly	Weekly monitor indicator for moisture.	Clean glassware with water, acetone, and acetonitrile. Repack with sicapent, glass wool, and ascarite.
Methane oven	2 years	N/A	Remove from the heating block, disassemble, and clean. Repack with a mixture of 4 Å molecular sieves/Shimalite-Ni (~1:1, 0.5 g).
Target gas	Yearly	Test the gas flow from the cyclotron through the module.	Check the flow rate of delivered gas from the cyclotron into the module. Adjust if necessary.
GM detectors	Yearly	Monitor activity from each run performed on the unit.	Detectors calibrated in yearly PM.
Fritz	Yearly	N/A	Fritz replaced in the yearly PM.
Cooling trap	As needed	N/A	Replace cooling trap column if necessary – crack determined.

5. GE TRACERlab Fx2 C Pro Cleaning

Before all production or test runs on GE TRACERlab Fx2 C Pro, cleaning and conditioning of the unit occur. Below are the cleaning sequence and conditioning steps taken at the NYUGSoM Radiochemistry Facility. Please note that the helium gas dedicated to each module is never turned off, with a constant backpressure of at least 80 psi.

Cleaning Module: The automated module and hot cell are checked with a calibrated Geiger counter before entering. All solid-phase extraction cartridges and filters are removed and checked with a calibrated Geiger counter before the cleaning sequence commences. Waste bottles and exhaust bags are emptied, with visualization of the hot cell to check for spills. A leak check is performed on the recirculation loop, reaction vessel, methyl iodide column, and methyl triflate column using a leak detector (helium gas), as well as internal feedback from the program by pressurizing designated areas of the module with helium gas; if pressure reaches above 1.00 KPa, troubleshoot the cause of the leak. The liquid nitrogen dewar is filled, and the HPLC loop is backflushed with acetonitrile (10 mL), water (10 mL), acetonitrile (10 mL), then air (20 mL). An embedded and validated in-house time list consisting of three sequences for cleaning is then executed; a summary of the process is as follows (see **Figure 15** in manuscript):

<u>Pre-flush: manual clean and blockage check of automated module:</u> The production team performs a pre-flush check before executing the cleaning time list to check for any blockages and to time the delivery of materials into the dispensing hot cell (DHC).

The inlet to the HPLC line is removed and placed into a waste container. Deionized water (1 mL) is first added to V1, V2, and V3. In manual mode with the use of helium gas (turn on V18), the operator directs the pathway of water from V1 into the reaction vessel. The operator then moves the water from the reaction vessel through valves V8 and V7, past the fluid detector and into a beaker to collect waste. This process is repeated for V2 and V3. This entire

process is further repeated with the use of acetone, followed by acetonitrile. This manual pre-flush process allows the production team to check for blocks in the lines to and from the reaction vessel. Once complete, the HPLC inlet line is tied back into position 1 of the injection loop.

The formulation and delivery unit of the module is manually pre-flushed. This process uses 20 mL of deionized water to pass through the receiving (globe) flask into the formulation flask via valves V11 through V12. The formulation flask contents are then pushed through the delivery line into our DHC. Deionized water (1 mL each) is then added to V4, V5, and V6, passed through V11 and V12 into the formulation flask. Each wash is separately passed into the DHC. This process is then repeated with 200 proof ethanol.

Automatic cleaning - Sequence 1: reagent vials and reaction vessel clean: The following solvents (1 mL for V1 and V2, 2 mL for V3) are added into the glass holding units for four individual sub-sequences: 1 N hydrochloric acid (1st step), water (2nd step), acetone (3rd step), and acetonitrile (4th step). The in-house automated sequence allows the added solvents to clean all lines and glassware associated with the reaction side of the module, through the reaction vessel and pass through valves V7 (side opposite check valve, e.g. liquid side of module), V8, and rinsing the HPLC loop into waste.

Automatic cleaning - Sequence 2: formulation cleaning and sterilization: Deionized water is added to the receiving flask (20 mL) and to each of the glassware affiliated with valves V4 (5 mL), V5 (1 mL), and V6 (10 mL). The automated sequence allows for the cleaning of lines and valves affiliated with V11 and V12, along with V13 and the delivery line into the DHC. Once the deionized water sequence is complete, the module is loaded with 200-proof ethanol (identical amount as deionized water). The lines are then cleaned and sterilized. Additional time is given in the sequence to dry out lines with the use of helium gas.

Automatic cleaning - Sequence 3: reaction vessel drying: The final executed sequence used for cleaning the GE TRACERlab Fx2 C Pro dries out the reaction vessel, as well as all lines affiliated with the reaction vessel. This is achieved with the use of vacuum, heating, and passing helium gas over and through the reaction vessel. Stepwise heating under vacuum (1 kPa) from 65 °C to 90 °C allows for the efficient drying of the reaction vessel. This process also provides the production team with the ability to check and monitor temperature changes and isolate any issues that may occur before a production run is underway. The complete pre-flush and embedded in-house cleaning time list may take up to 50 min to complete.

6. Construction of Methyl Iodide Calibration Curve by HPLC

Preparation of methyl iodide standards

I. Primary and intermediate stock solutions: Weigh 1 mL of methyl iodide (approximately 2.50 g) into a tared 100 mL volumetric flask and dilute to volume with a diluent (80:20 acetonitrile: water, v/v) to prepare a primary stock solution at 25.0 mg/mL. Ensure that the solution is well-mixed and homogeneous. Next, prepare an intermediate stock solution by transferring 1 mL of the primary stock solution into a 50 mL volumetric flask and dilute to volume with the same diluent to obtain a 500 μg/mL solution .

II. Reference standard solutions: Prepare the following 5 mL reference standard solutions in 7.5 mL vials by pipetting the appropriate volume of primary stock solution and the remaining volume of diluent.

3 μg/mL: Add 30 μL of intermediate stock solution and 4.970 mL of 80:20 acetonitrile: water (v/v).

10 μg/mL: Add 100 μL of intermediate stock solution and 4.900 mL of 80:20 acetonitrile: water (v/v).

15 μg/mL: Add 150 μL of intermediate stock solution and 4.850 mL of 80:20 acetonitrile: water (v/v).

25 μg/mL: Add 250 μL of intermediate stock solution and 4.750 mL of 80:20 acetonitrile: water (v/v).

60 μg/mL: Add 600 μL of intermediate stock solution and 4.400 mL of 80:20 acetonitrile: water (v/v).

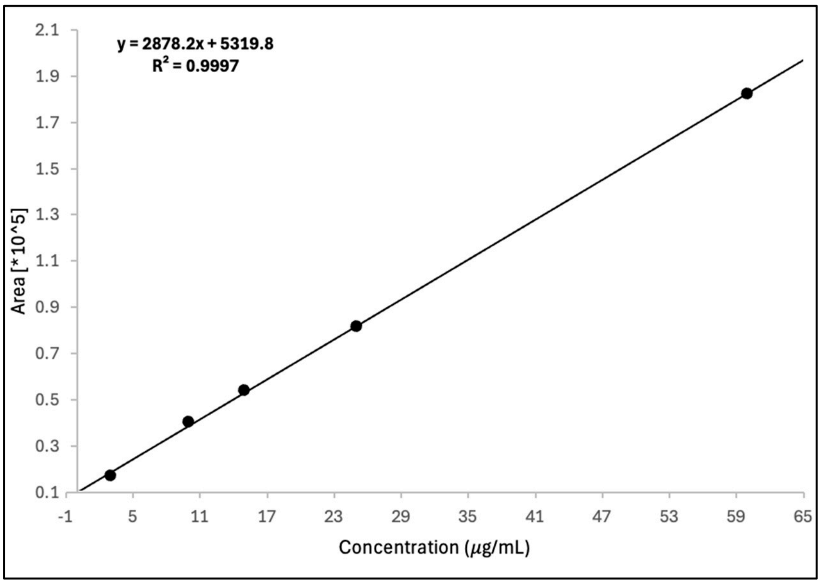

Figure S5. Calibration curve for methyl iodide.

Methyl iodide calibration curve

The calibration curve for methyl iodide was constructed from five replicates per reference standard (3-60 μg/mL), yielding an excellent R^2 = 0.999 (see **Figure S5**). This curve has demonstrated a linear response suitable for quantification of methyl iodide to aid in our determination of the isotopologue mass of methyl iodide found in both intrinsic and radioactive test runs.

7. References

1. General Electric. TRACERlab FXc Operator Guide. **2004**.
2. General Electric. TRACERlab FXc Service Manual. **2004**.
